# Addressing the credibility crisis in Mendelian randomization

**DOI:** 10.1186/s12916-024-03607-5

**Published:** 2024-09-11

**Authors:** Stephen Burgess, Benjamin Woolf, Amy M. Mason, Mika Ala-Korpela, Dipender Gill

**Affiliations:** 1 Medical Research Council Biostatistics Unit, University of Cambridge, Cambridge, UK; 2 Cardiovascular Epidemiology Unit, Department of Public Health and Primary Care, University of Cambridge, Cambridge, UK; 3 School of Psychological Science, University of Bristol, Bristol, UK; 4 MRC Integrative Epidemiology Unitat the , University of Bristol, Bristol, UK; 5 Victor Phillip Dahdaleh Heart and Lung Research Institute, University of Cambridge, Cambridge, UK; 6 Systems Epidemiology, Faculty of Medicine, Research Unit of Population Health, University of Oulu and Biocenter Oulu, Oulu, Finland; 7 NMR Metabolomics Laboratory, School of Pharmacy, Faculty of Health Sciences, University of Eastern Finland, Kuopio, Finland; 8 Department of Epidemiology and Biostatistics, School of Public Health, Imperial College London, London, UK; 9 Sequoia Genetics, London, UK

**Keywords:** Causal inference, Genetic epidemiology, Instrumental variables, Evidence synthesis, Risk of bias, Bias evaluation

## Abstract

**Background:**

Genome-wide association studies have enabled Mendelian randomization analyses to be performed at an industrial scale. Two-sample summary data Mendelian randomization analyses can be performed using publicly available data by anyone who has access to the internet. While this has led to many insightful papers, it has also fuelled an explosion of poor-quality Mendelian randomization publications, which threatens to undermine the credibility of the whole approach.

**Findings:**

We detail five pitfalls in conducting a reliable Mendelian randomization investigation: (1) inappropriate research question, (2) inappropriate choice of variants as instruments, (3) insufficient interrogation of findings, (4) inappropriate interpretation of findings, and (5) lack of engagement with previous work. We have provided a brief checklist of key points to consider when performing a Mendelian randomization investigation; this does not replace previous guidance, but highlights critical analysis choices. Journal editors should be able to identify many low-quality submissions and reject papers without requiring peer review. Peer reviewers should focus initially on key indicators of validity; if a paper does not satisfy these, then the paper may be meaningless even if it is technically flawless.

**Conclusions:**

Performing an informative Mendelian randomization investigation requires critical thought and collaboration between different specialties and fields of research.

## Background

Mendelian randomization is an epidemiological technique that exploits the properties of genetic variants to address causal questions about the potential effect of an exposure on an outcome [1, 2]. Mendel’s laws of heritability mean that, conditional on parental genotype, genetic variants should only be associated with traits that they influence [3, 4]. Given a well-mixed population, the same property should hold at the population level [5]. Empirical investigations have shown that genetic associations with unrelated traits estimated in population-based cohorts are no stronger than would be expected due to chance alone [6, 7]. This suggests a generic strategy for testing the causal effect of any exposure on any outcome by the following steps:

1. Find genetic variants that are predictors of the exposure
2. Test whether these genetic variants associate with the outcome

The simplicity and universality of the approach is appealing [8]. Analogously to a randomized trial, inferences are made not by application of clever statistical methodology, but by exploiting random variation [9, 10]—although in the case of Mendelian randomization, this is naturally-occurring randomization rather than random allocation by a trialist [11]. However, such a simple recipe cannot provide reliable causal inferences without thoughtful application.

The Mendelian randomization approach relies on the gene–environment equivalence principle [12]. This states that selected genetic variants influence an environmental (that is non-genetic) exposure equivalently to a proposed intervention that changes the population distribution of the exposure. In practice, there are often differences between the effect of a genetic variant and a proposed intervention in terms of mechanism, magnitude, timing, and duration that imply downstream consequences are not exactly equivalent [13, 14]. The principle can be restated to require that genetic associations are informative about the presence, direction, and (to a more limited extent) the size of the effect on the outcome resulting from an intervention in the exposure.

The availability of data from genome-wide association studies (GWAS) has enabled Mendelian randomization analyses to be performed at an industrial scale [15, 16]. In particular, it has enabled two-sample summary data Mendelian randomization investigations [17]: “two-sample” indicates that genetic associations with the putative exposure and outcome come from different datasets; “summary data” indicates that analyses are performed using genetic association estimates—beta-coefficients and standard errors representing associations of the respective variants with the exposure and outcome—rather than individual-level data [18, 19]. Such association data have been released for many large consortia and biobanks [20, 21]. Anyone with access to the internet can download genetic associations with risk factors and disease outcomes and use these to implement Mendelian randomization methods [22]. Indeed, such applications of Mendelian randomization have great advantages: they are able to use large datasets published by GWAS consortia, and analyses can be made fully transparent and replicable.

However, particularly in the age of artificial intelligence, such analyses are arguably too accessible. Web-based tools have been created that simplify the task of the analyst to simply choosing the exposure and outcome—the automated analysis is performed at the touch of a button [23]. Mendelian randomization has become an easy target for researchers who are incentivized to publish as often as they can, as well as to predatory journals which are willing to publish such articles. While the two-sample summary data approach has led to many insightful papers, it has also fuelled an explosion of poor-quality Mendelian randomization publications, which threatens to overwhelm the capacity of qualified reviewers and undermine the credibility of the whole approach.

The guidance in this article is written to help those who want to write meaningful Mendelian randomization papers, as well as to journal editors and reviewers seeking to triage and identify low-quality submissions. There is already plentiful guidance on performing and reporting Mendelian randomization investigations [24], including the Strengthening the Reporting of Observational Studies in Epidemiology using Mendelian Randomization (STROBE-MR) guidelines [25]; we would encourage journals to insist that authors complete the checklist based on these guidelines at initial submission. This is important to ensure analyses are performed accurately and to avoid errors, such as mistakes in allele harmonization [26]. However, a Mendelian randomization investigation may be perfectly written and follow these guidelines to the letter—and yet the whole study may be completely useless.

We focus here on two-sample Mendelian randomization analyses using established methods for the analysis of summarized data. Advanced methods, such as non-linear analyses [27], cross-generational analyses [28], and time-varying analyses [29], require additional assumptions and detailed considerations that could potentially lead to biased estimates if violated [30–32]. Such methods are outside the scope of this paper. However, the considerations discussed here about instrument selection, instrument validity, and interpretation are foundational, and also apply to such applications.

**Fig. 1:**
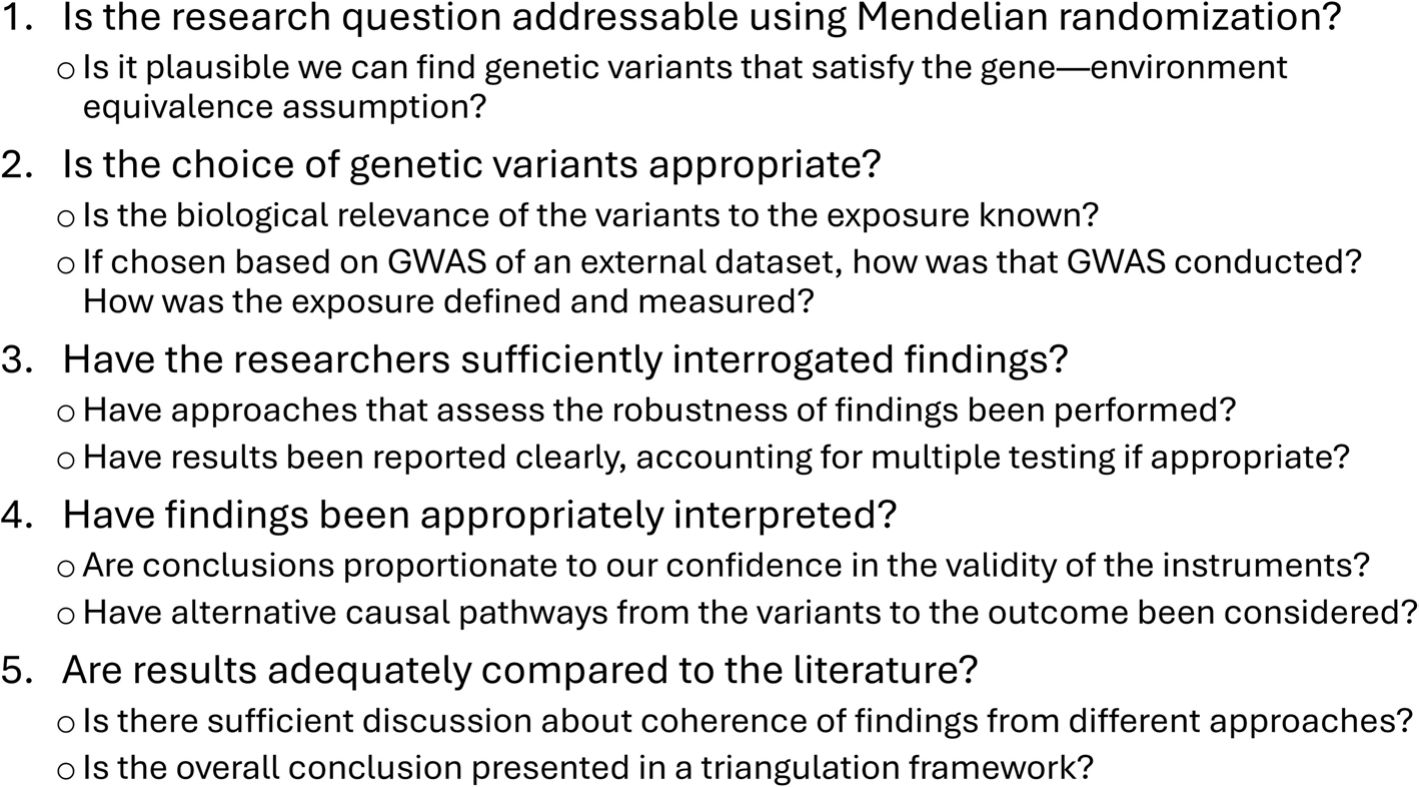
Key considerations when assessing the credibility of a Mendelian randomization investigation

We consider five common pitfalls in conducting a reliable Mendelian randomization investigation: (1) inappropriate research question, (2) inappropriate choice of variants as instruments, (3) insufficient interrogation of findings, (4) inappropriate interpretation of findings, and (5) lack of engagement with previous literature. We present a short list of relevant questions relating to these points in Fig. 1 for authors to consider. While not as comprehensive as the STROBE-MR guidelines, it is more succinct and focuses on the key critical judgements that are required to assess the reliability of an investigation. It should be particularly valuable not just to authors, but also to reviewers and editors, and indeed, to eventual readers wanting to evaluate the quality of evidence provided by a Mendelian randomization publication.

### Inappropriate research question

The instrumental variable assumptions [33] require that any genetic variant used in a Mendelian randomization investigation as an instrument must:

1. Be associated with the exposure (relevance)
2. Not be associated with the outcome via a confounding pathway (exchangeability)
3. Have no direct effect on the outcome, only potentially an indirect effect via the exposure (exclusion restriction) [34]

Only the first of these assumptions can be verified based on data. The other two assumptions cannot be formally tested and must be justified either on the basis of scientific understanding, or empirically supported based on the application of statistical methods [24].

**Fig. 2:**
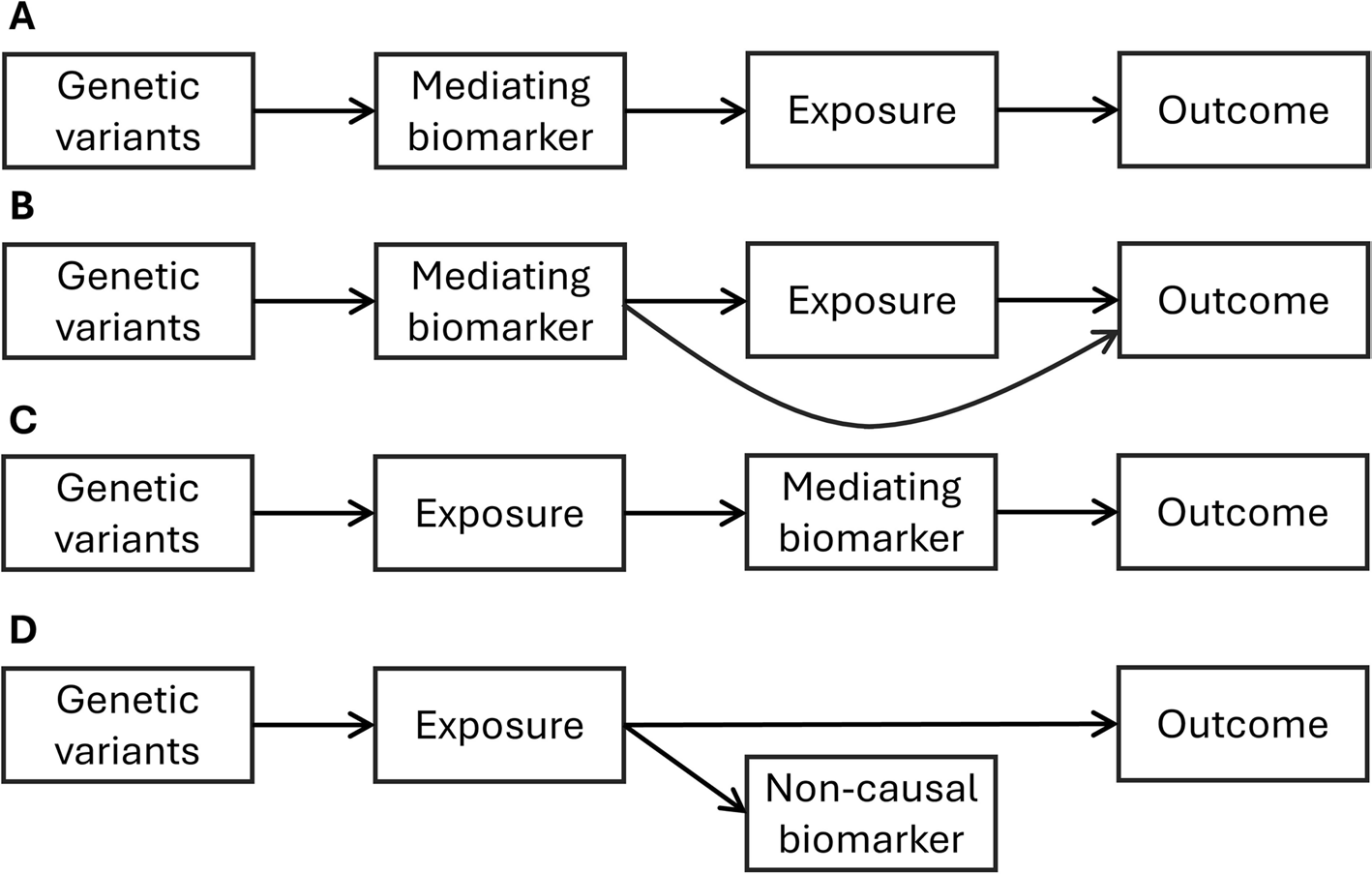
Genetic associations with an exposure variable that is downstream of a mediating biomarker (diagrams A and B), or has a downstream effect on either a mediating biomarker (diagram C) or a non-causal biomarker (diagram D). In case A, the only causal pathway from the genetic variants to the outcome passes via the exposure; hence, this is an example of “vertical pleiotropy”, and the genetic variants are valid instruments. In case B, there is a causal pathway from the genetic variants to the outcome that does not pass via the exposure; hence, this is an example of “horizontal pleiotropy”, and the genetic variants are not valid instruments. In cases C and D (which also represent “vertical pleiotropy”), Mendelian randomization analyses should be conceptualized in terms of the exposure (the putative causal trait), even if measured genetic associations are expressed in terms of the biomarker. Diagram D is likely to represent the situation between genetic variants in the *IL6R* gene region, interleukin 6 signalling (exposure), and C-reactive protein (non-causal biomarker). C-reactive protein is likely to be a non-causal biomarker when considering the effect of interleukin 6 receptor inhibition on coronary heart disease [36]

These assumptions require the genetic variants to be specific in how they affect the exposure—there cannot be pleiotropic associations with variables on alternative causal pathways to the outcome. Associations with variables on the causal pathway from the genetic variants to the outcome via the exposure (sometimes called “vertical pleiotropy”) are allowed; associations with variables on alternative causal pathways (sometimes called “horizontal pleiotropy”) are not [35] (Fig. 2).

For some exposures, it is implausible that there are genetic variants that influence the exposure in a way that meets these requirements. A paradigmatic example of such an exposure is “use of chopsticks”—if a researcher found genetic predictors of chopstick use in a Western population, the likely explanation would be that the variants reflect demographic or socioeconomic status, rather than a biological mechanism that affects cutlery choice [37]. Such variants would be invalid instrumental variables: first, they would be subject to population stratification, and second, even if population stratification could be addressed, they would be associated with other traits and behaviours that are more common in chopstick users. As such, a Mendelian randomization study supposedly finding evidence of an effect of chopsticks use would have to show that this effect is not attributable to the many other social and cultural factors that likely differ between the genetically defined population groups.

Another implausible exposure for use in Mendelian randomization is pollution levels [38]. Again, it is implausible that there are particular genetic variants that affect exposure to air pollution. If genetic predictors of air pollution are found, it is likely that these are markers of social status rather than representing intrinsic biological mechanisms. In some large datasets, such as UK Biobank, air pollution is not measured at an individual level, but inferred based on home address [39]. This reinforces the concern that such an analysis is actually evaluating social status, not air pollution in any specific way. Another category of implausible exposures for Mendelian randomization is gut microbiota [40]. It is implausible that there are particular genetic variants that have specific effects on individual gut microbiome species. While some genetic predictors of gut microbiota have been found, they are located in highly pleiotropic gene regions, such as the *ABO* gene region [41]. Just because a GWAS has found genetic predictors of a trait does not imply that the trait is an appropriate exposure for a Mendelian randomization investigation, nor that the genetic predictors represent valid genetic instruments.

If an exposure is externally or environmentally determined, or variation in the exposure is influenced purely by social and cultural factors rather than intrinsic biological mechanisms, then it is unlikely that effects of the exposure can be reliably interrogated in a Mendelian randomization design. Such traits are more likely to be subject to bias from population stratification, non-random mating patterns, and dynastic effects (that is inter-generational effects, such as when the parental genotype directly influences the offspring exposure or outcome) [42].

A counter-example to this is alcohol consumption. While alcohol consumption is partially determined by personal and environmental factors, there are biological mechanisms influencing the metabolism of alcohol that affect consumption levels, as well as exposure to alcohol in the bloodstream. Genetic variants in key regulators of these mechanisms are potential instruments for understanding the downstream effects of alcohol consumption [43]. However, care is still required to appropriately perform and interpret such analyses; we follow up this example in further sections.

Researchers should be aware that not all causal questions can appropriately be addressed in a Mendelian randomization paradigm. Journal editors and reviewers should use their judgement to rapidly decide whether a question can plausibly be addressed by Mendelian randomization based on the abstract (or even the title) alone: is it plausible that there exist genetic variants such that the gene–environment equivalence principle holds? That is, are there likely to be genetic variants that affect the exposure in a way equivalent to the (possibly hypothetical) intervention implied by the causal question under investigation? If this is unlikely, then the investigation, even if perfectly implemented and reported, does not provide reliable evidence to address the causal question of interest.

### Inappropriate choice of variants as instruments

The instrumental variable assumptions require that any causal pathway from the genetic variants to the outcome passes via the exposure under investigation. This is more plausible if the genetic variants are located in a gene region with known functional or biological relevance to the exposure [44–46]. It is less plausible for exposures that the genetic variants influence indirectly via complex causal pathways, such as educational attainment. For example, genetic variants in the *UGT1A1* gene region that encodes an enzyme regulating the synthesis of bilirubin are more plausible instruments than variants in gene regions that are not functionally related to bilirubin, or whose function is unknown [47]. Genetic variants in the *ALDH2* and *ADH1B* gene regions are known to relate to alcohol metabolism, and hence are plausible instruments to investigate the effect of alcohol consumption [48]. If gene regions with biological relevance to the exposure are not known, Mendelian randomization can provide some evidence on the causal relevance of the exposure, but additional caution is required [49].

For a given gene region, genetic variants should be chosen based on their biological relation to the causal risk factor of interest, as far as is possible. This includes proximity to the relevant gene and functional effects on regulation of the gene or its downstream protein. For example, when investigating the effect of angiotensin converting enzyme (ACE) on risk of Alzheimer’s disease, use of variants in the *ACE* gene region predicting tissue-specific gene and protein expression likely increases their plausibility as valid instruments for pharmacological perturbation of ACE at the relevant biological site [50]. In some cases, the same variant may be the lead signal for circulating protein levels, gene expression in the most relevant tissue, and levels of a downstream risk factor. In other cases, these approaches may identify different variants [51]. If these differ, careful consideration is needed to select the variant(s) that best mimic the intervention of interest.

Biological mechanisms affecting many exposures are not known. In such cases, genetic variants used as instruments may be selected solely based on their statistical association with the exposure. Such analyses are often still valuable, in that they provide a source of evidence supporting or refuting a causal effect of the exposure on the outcome. The strength of evidence provided depends on our confidence in the validity of the genetic variants as instruments. Testing genetic associations with potential confounders can provide empirical evidence supporting the validity of the variants as instruments, as can other statistical approaches, such as the application of pleiotropy-robust methods [52].

Researchers should prioritize investigating exposures using variants in gene regions that are biologically related to the exposure where possible. Journal editors and reviewers should look for a justification as to why the genetic variants in a given analysis were chosen. If this is absent, or if genetic variants are purely chosen on statistical grounds, then findings will generally be less authoritative and require a greater degree of statistical assessment.

### Insufficient interrogation of findings

While the exact analysis plan will depend on the specifics of the question under investigation, availability of valid instruments, data quality, and so forth, one recommended generic strategy for conducting Mendelian randomization analyses is as follows. First, if there are biologically informed candidate instruments, the primary analysis should be based on these variants. Second, if there are no biologically informed candidate instruments, an initial liberal analysis based on a wide range of variants is recommended. Finally, results should be interrogated further to investigate robustness to a variety of factors [24]. A null finding in a liberal analysis that includes potentially pleiotropic variants is likely to reflect a true null relationship; it is more likely that bias will lead to a false positive finding than a false negative finding [53]. However, false negative results can be just as harmful to science. Absence of evidence does not always mean evidence of absence, particularly if the analysis is underpowered, unspecific, or poorly designed.

There are many approaches for the interrogation of findings (see reference [24] for more details), including examining genetic associations with potentially pleiotropic variables [54], testing against positive and/or negative controls [55], colocalization (particularly when the finding is based on a single gene region) [56], use of pleiotropy-robust methods (particularly when the finding is based on variants from several gene regions) [52], investigation in subgroups of the population [57] (although noting such stratification can lead to collider bias [58]), investigation with a subset of variants, and multivariable Mendelian randomization [59]. No single sensitivity analysis approach is foolproof [60], and all approaches make their own assumptions. A causal effect may be present even if one or more approach does not provide supportive evidence of a causal effect (or equally, a causal effect may be absent even if one or more approach supports a causal effect). In many cases, the evidence will be equivocal; there may be evidence supporting a causal effect, but this evidence may not be fully consistent across all analyses. If there is inconsistent evidence, then it is important that results are reported clearly, without undue emphasis on significant findings. Similarly, if multiple hypotheses are tested by the investigators, this should be accounted for when interpreting findings.

As an example, the robustness of Mendelian randomization analyses with alcohol as an exposure has been tested in several ways. Analyses in East Asian populations have typically used variants in the *ALDH2* and *ADH1B* gene regions as instruments [61]. These investigations have exploited a further natural experiment by conducting analyses separately for men and women. Genetic associations with disease outcomes would not be expected in East Asian women as their alcohol consumption levels are much lower than those of men. East Asian women represents a negative control population, and null associations in women but positive associations with men have been observed for oesophageal cancer [62] and blood pressure [63]. In European-descent populations, similar findings have been observed using a variant in the *ADH1B* gene region only and using a wider range of genetic predictors of alcohol consumption [64]. Consistent results for many outcomes have been observed across a range of robust methods, including MR-Egger, weighted median, and MR-PRESSO methods [65]. Multivariable analyses have also been conducted adjusting for smoking behaviour, as genetic predictors of alcohol may have pleiotropic effects on smoking intensity [66].

Researchers should perform a range of approaches to investigate the robustness of findings. The reported level of confidence in conclusions should be dependent on the consistency of these results. Journal editors and reviewers should be suspicious of selective reporting of significant findings, particularly when approaches to assess the validity of findings have either not been reported, or indicate lack of support for a causal effect.

### Inappropriate interpretation of findings

We have hereto assumed that the exposure measured in the Mendelian randomization analysis is the true causal agent affecting the outcome. However, this may not be the case. It is possible that a version of the gene–environment equivalence principle is true, but not for the measured exposure. It may be that the measured exposure is a biomarker that acts as a proxy measure of the true causal mechanism of action (Fig. 2).

As an example, genetic variants in the *IL6R* gene region are associated with levels of both interleukin 6 and C-reactive protein. This is plausibly an example of vertical pleiotropy, as the association with C-reactive protein is potentially a downstream consequence of the effect of interleukin 6 receptor signalling [36]. If we use genetic variants in the *IL6R* gene region in a Mendelian randomization analysis investigating the effects of interleukin 6 receptor signalling, we should come to the same conclusion whether our nominal biomarker for selecting and weighting instruments is levels of interleukin-6 receptor or levels of C-reactive protein [67]. Our estimate may be expressed in terms of change in genetically predicted interleukin-6 receptor levels or genetically predicted C-reactive protein levels, but it is the choice of variants that determines the causal question that is being addressed, not the biomarker used to select instruments for the exposure. As a further hypothetical example, suppose that we performed a Mendelian randomization analysis using genetic predictors of left leg mass. Would we be confident that any finding was truly attributable to an effect of left leg mass as opposed to adiposity or muscle mass more generally?

A related issue, particularly for binary exposures, is that genetic variants increase liability to the exposure, but do not necessarily increase the exposure [68]. For example, most individuals having genetic variants associated with increased schizophrenia risk do not themselves have clinically diagnosed schizophrenia [69]. Genetic variants that predispose individuals to increased alcohol consumption do not increase exposure to alcohol in populations of non-alcohol drinkers. Genetic variants shown to predispose individuals to greater COVID-19 risk did not increase exposure to COVID-19 in pre-pandemic datasets.

Mendelian randomization is serendipitous in nature; we exploit what is available. We cannot control which genetic variants are available for our analysis, or what these genetic variants do. The gene–environment equivalence principle requires to first understand how the genetic variants operate, and express our causal question in terms of the tools that are available. This implies that a simple conclusion statement such as “the exposure has a causal effect on the outcome” may not be appropriate.

Returning to the example of alcohol, genetic variants that increase alcohol consumption may have effects relating to social aspects of alcohol consumption as well as biological aspects. Those who drink more alcohol in Western societies are likely to spend more time in licenced establishments, and potentially have greater exposure to environmental tobacco smoke. Another complication is distinguishing between alcohol consumption and exposure to high alcohol levels. For caffeine, genetic associations with coffee consumption and circulating plasma caffeine levels are not all concordant. This can be explained as some genetic variants that increase caffeine metabolism lead to lower circulating caffeine levels, but greater coffee consumption, as fast caffeine metabolizers tend to consume more coffee to get the same physiological response [70]. Variants in the *ALDH2* and *ADH1B* gene regions affect alcohol consumption via different biological pathways. While the rs671 variant in *ALDH2* decreases alcohol consumption, it impairs the metabolism of alcohol, meaning that carriers who drink alcohol have greater exposure to acetaldehyde, a known carcinogen [71]. Hence, the associations of the rs671 variant may be misinterpreted if investigators focus on associations with alcohol consumption level. Correct interpretation of Mendelian randomization analyses requires appreciation of the broad social context of alcohol consumption and understanding of the biological effects of the variants.

Researchers should think carefully about the identity of the underlying causal risk factor or mechanism evaluated in their analysis; this may differ from the measured variable used as the exposure. Journal editors and reviewers should be sceptical about strong causal claims. Jumps of logic from factual statements such as “genetic predictors of the exposure were associated with the outcome” (or equivalently “genetically predicted exposure levels were associated with the outcome”) to subjective inferences such as “therefore, we believe the exposure is a cause of the outcome” should only be made when they can be justified [72]. If it is implausible that an exposure could be altered in a specific way by a genetically regulated mechanism, then it may be that the nominal exposure is a biomarker for a wider mechanism, not the literal causal risk factor.

### Lack of engagement with previous literature

Mendelian randomization cannot by itself demonstrate or prove the existence of a causal effect. Indeed, the aim of a Mendelian randomization investigation is often to provide supportive or suggestive evidence to encourage further research, including the establishment of a randomized trial. As such, it is important to weigh evidence from Mendelian randomization against that from other approaches, including epidemiological data, trial findings, and basic science experiments. Triangulation is a framework for evidence synthesis that considers evidence from various sources that make different assumptions, and hence the validity of these assumptions will be orthogonal [60, 73]. Evidence from different approaches making different assumptions can provide a more compelling case for a causal effect, or can help enhance the specificity of evidence. By showing that evidence for a causal effect is stronger or weaker in certain circumstances (such as different populations, different times, or different subgroups), we can improve our understanding of the causal mechanism.

In the case of alcohol, while there are no large-scale long-term randomized trials investigating the impact of alcohol consumption on disease outcomes [74], there are randomized trials exploring the effects of drinking alcohol in the short term, and many mechanistic studies into the effects of alcohol. While several observational epidemiological studies have shown lower risk of cardiovascular disease amongst light drinkers compared to non-drinkers [75], Mendelian randomization analyses have not supported evidence of a protective effect of increased alcohol at any level of alcohol consumption [61, 76]. A Mendelian randomization investigation into the effect of alcohol consumption should explore reasons for discrepancies from results from conventional observational analyses. A potential explanation is that the non-drinker category contains both never-drinkers and former-drinkers, and the observational elevated risk in non-drinkers is due to former-drinkers.

Researchers should compare findings from their Mendelian randomization investigation to those from lab-based experiments, functional genomic studies, observational epidemiological associations, and clinical trials. Results should be appraised in a triangulation framework indicating the extent to which they strengthen or weaken the evidence for a causal effect of the exposure. Journal editors and reviewers should hold authors to high standards and ensure that findings are adequately compared to those from previous Mendelian randomization investigations and other approaches.

## Conclusions

Mendelian randomization can be applied in an uncritical, algorithmic way to obtain findings and generate publications [77]. Policing such outputs is an impossible task requiring far more resources than it takes to create the publications, and the onus should be on authors to perform thoughtful and well-justified analyses. Journal editors at reputable journals should be able to spot low-effort submissions without wasting precious peer review resources. Reviewers should focus not only on whether technical aspects of a submission are present, but also on key indicators that require critical judgement: whether the causal question can plausibly be addressed by Mendelian randomization, whether the choice of variants is justified, whether there has been sufficient interrogation of findings (assessment of internal validity), whether any inferred causal interpretation is appropriate (assessment of external validity), and how this finding supports or refutes aspects of the wider literature. Performing such an investigation requires close collaboration between those with biological, clinical, sociological, genetic, and statistical expertise to understand the plausibility of the assumptions and to perform and interpret analyses appropriately.

## Data Availability

Not applicable; this manuscript does not contain primary data or analyses.

## Abbreviations

ACE: Angiotensin converting enzyme
GWAS: Genome-wide association study/studies
STROBE-MR: Strengthening the Reporting of Observational Studies in Epidemiology using Mendelian Randomization

## Data availability

No datasets were generated or analysed during the current study.
